# PD-1 inhibitor combined with chemotherapy for first-line treatment of esophageal squamous cell carcinoma patients with distant metastasis: a real-world retrospective study

**DOI:** 10.3389/fimmu.2024.1353445

**Published:** 2024-03-21

**Authors:** Loulu Gao, Lin Tang, Jieqiong Peng, Zixuan Hu, Jing Yang, Bo Liu

**Affiliations:** ^1^ School of Clinical Medicine, Weifang Medical University, Weifang, China; ^2^ Department of Oncology, Shandong Cancer Hospital and Institute, Shandong First Medical University and Shandong Academy of Medical Sciences, Jinan, China; ^3^ Department of Oncology, Shandong First Medical University and Shandong Academy of Medical Sciences, Jinan, China

**Keywords:** PD-1 inhibitors, chemotherapy, esophageal squamous cell carcinoma, first-line treatment, real-world study

## Abstract

**Background:**

The aim of this study was to evaluate whether the efficacy and safety of PD-1 inhibitors combined with chemotherapy in the treatment of patients with esophageal squamous cell carcinoma (ESCC) with distant metastasis in the real world are as effective and safe as in clinical trials.

**Patients and methods:**

From July 2019 to July 2023, a total of 422 patients with distant metastasis of ESCC were included and divided into the PD-1 inhibitor combined chemotherapy group (PC group) and the chemotherapy alone group (C group) according to the treatment regimen. There were 278 patients in the PC group and 144 patients in the C group. The primary endpoint of this study was progression-free survival (PFS), while secondary endpoints included objective response rate (ORR), disease control rate (DCR), overall survival (OS), and safety.

**Results:**

The objective response rate (ORR) and disease control rate (DCR) of the PC group were 44.60% (124/278) and 91.00% (253/278), respectively, which were 18.9% and 3.5% higher than those of the C group. The median PFS and median OS of the PC group were significantly better than those of the C group (median PFS: 6.5 vs. 5.5 months, *P* < 0.001; median OS: 16.6 vs. 13.9 months, *P* = 0.002). Further univariate and multivariate Cox analysis showed that the Eastern Cooperative Oncology Group performance status (ECOG PS) score and the number of metastatic sites were potential predictors of PFS in PC patients. The combination of PD-1 inhibitors with cisplatin and paclitaxel (TP) was more beneficial for patients with PFS compared to the combination of cisplatin and fluorouracil (PF). Furthermore, the presence of bone metastasis, body mass index (BMI), and lymphocyte-to-monocyte ratio (LWR) before treatment may be potential predictive factors for patient OS. The adverse reactions that occurred in the PC group can be tolerated or alleviated after both prevention and active treatment.

**Conclusions:**

The combination of PD-1 inhibitors and chemotherapy as first-line treatment for ESCC patients with distant metastasis still has good efficacy and safety compared to clinical trials in the real world.

## Introduction

Esophageal cancer is a common gastrointestinal tumor, the seventh most common cancer, and the sixth most common cause of cancer-related death globally (1). More than two-thirds of esophageal cancer patients are diagnosed with advanced or metastatic disease (2). Esophageal cancer consists of two main histological types, including esophageal squamous cell carcinoma (ESCC) and esophageal adenocarcinoma (EAC). ESCC is the main histological subtype in Asia, accounting for approximately 90% of all esophageal cancer cases, and more than half of global ESCC cases occur in China (3). For advanced or metastatic ESCC, the standard first-line chemotherapy regimen is mainly platinum combined with fluorouracil (PF) or paclitaxel (TP). The clinical benefits are still limited, and the median overall survival (OS) of patients is generally around one year, with no further breakthroughs (4–6).

Multiple clinical trials have shown that PD-1 inhibitors in immunotherapy drugs have good clinical efficacy in the first-line treatment of advanced esophageal cancer (7–12). For example, in the KEYNOTE-590 study, pembrolizumab plus chemotherapy improved median OS by more than 5 months versus placebo plus chemotherapy (13.9 vs. 8.8 months; HR: 0.57, 95% CI: 0.43-0.75; *P* < 0.0001) and median progression-free survival (PFS) (7.5 months vs. 5.5 months; HR: 0.51, 95% CI: 0.41-0.65; *P* < 0.0001). In addition, the median OS was nearly 3 months longer in all randomized patients (12.6 months vs. 9.8 months; HR: 0.72, 95% CI: 0.60-0.88; *P* = 0.0006), and the median PFS was 0.5 months longer (6.3 months vs. 5.8 months; HR: 0.65, 95% CI: 0.54-0.78; *P* = 0.0001). According to the findings of this study, pembrolizumab became the first PD-1 inhibitor approved for the first-line treatment of advanced esophageal cancer, breaking the bottleneck period for advanced ESCC treatment (7). Following that, several phase III trials, including CHECKMATE-648, ESCORT-1st, ORIENT-15, RATIONALE 306, and ASTRUM-007, demonstrated the efficacy of immunotherapy combined with chemotherapy in the first-line treatment of advanced esophageal cancer (7–11).

For the above clinical trials, they are usually conducted in a controlled environment with strict selection and standardization of the study population and treatment regimen. In contrast, real-world research can provide data on treatment efficacy and survival status in actual clinical practice. Currently, there is a lack of real-world data analysis for advanced first-line treatment of patients with distant metastasis of ESCC. This article retrospectively analyzes the efficacy and safety of PD-1 inhibitors combined with chemotherapy in the treatment of patients in practical applications and further explores which factors may affect survival prognosis. Comparing the results under clinical trial conditions, this consideration of actual treatment situations can provide a more comprehensive and realistic evaluation of treatment effectiveness, provide more comprehensive treatment guidance, and better select personalized treatment plans for patients. Real-world research can provide more data support and scientific basis for clinical applications, accumulate more clinical evidence, and provide a more reliable basis for decision-making. In addition, this study evaluates the impact of this treatment regimen on patient survival outcomes, providing more comprehensive data support for clinical practice.

## Study design and patients

**Figure 1:**
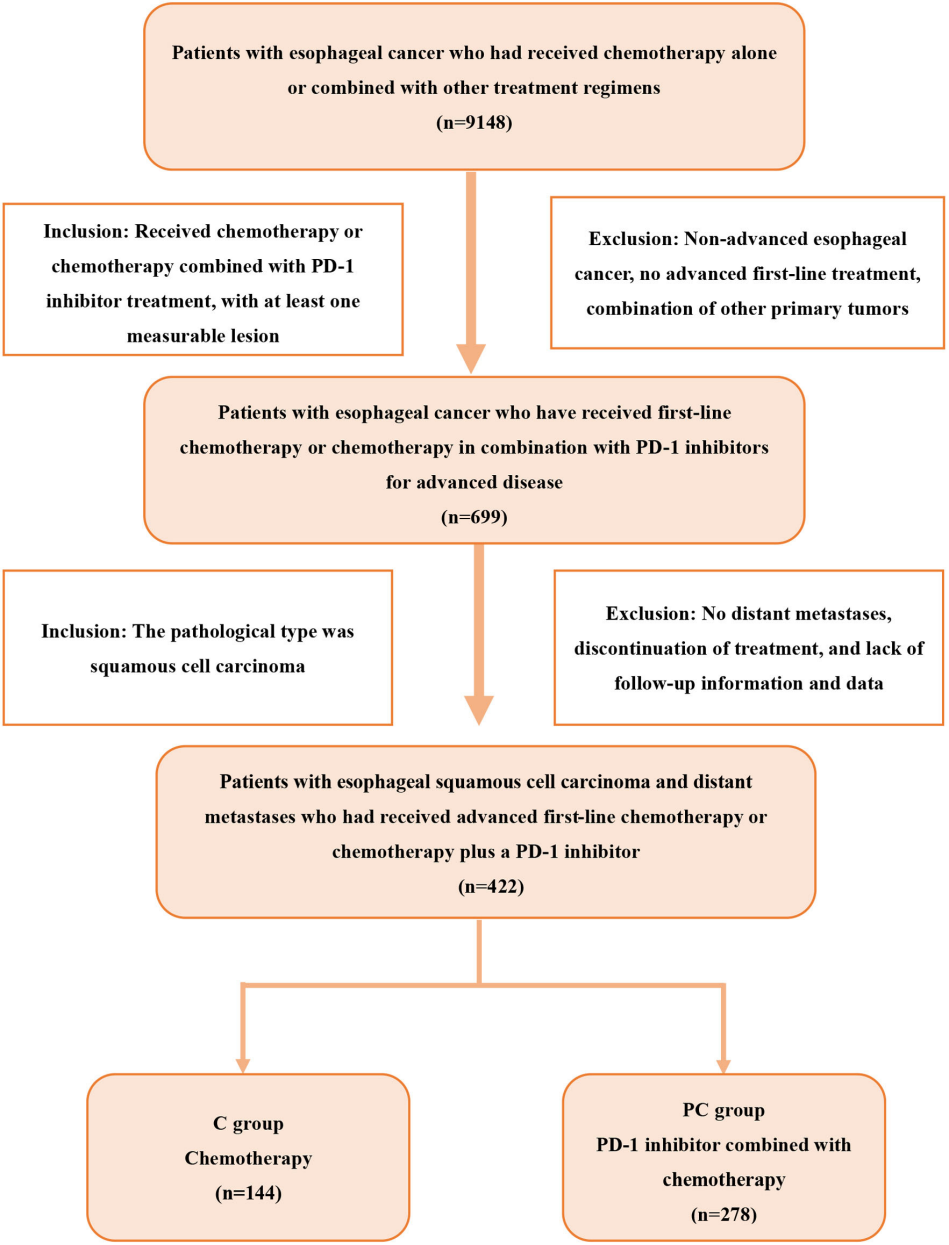
Flowchart of the screening procedure. PD-1, programmed cell death protein 1.

We retrospectively analyzed patients with esophageal cancer who were treated at Shandong Cancer Hospital from July 2019 to July 2023 and met the following criteria: 1) ESCC confirmed by histology or cytology; 2) presence of distant metastatic disease; 3) no previous advanced systemic therapy; 4) at least one measurable lesion according to Response Evaluation Criteria in Solid Tumors (RECIST) version 1.1. The exclusion criteria were as follows: 1) the presence of other primary malignant tumors; 2) the presence of underlying diseases that could affect the assessment of efficacy and safety, including autoimmune diseases, severe cardiovascular diseases, or diseases related to liver dysfunction, renal dysfunction, and thyroid dysfunction. According to the treatment, patients were divided into two groups: the PD-1 inhibitor combined with chemotherapy group (PC group) and the chemotherapy group (C group). The screening process is shown in **Figure 1**. All patients were followed up until December 2023, or death.

In this study, we retrospectively collected information on patient characteristics, survival status, treatment outcomes, and clinical pathological characteristics. We also collected data on complete blood cell count, serum lactate dehydrogenase (LDH) levels, and serum albumin levels before starting treatment. Additionally, we calculated the neutrophil-to-lymphocyte ratio (NLR), platelet-to-lymphocyte ratio (PLR), and lymphocyte-to-monocyte ratio (LMR), using the median values of NLR, PLR, and LMR as cut-off points. Patient heights and weights before treatment were also collected, and the body mass index (BMI) (kg/m^2^) was categorized into four levels based on the characteristics of Chinese adults: underweight (<18.5 kg/m^2^), normal weight (18.5-23.9 kg/m^2^), overweight (24.0-27.9 kg/m^2^), and obesity (>=28.0 kg/m^2^).

In the study, PD-1 inhibitors used by patients included camrelizumab, sintilimab, pembrolizumab, toripalimab, tislelizumab, and nivolumab. The chemotherapy regimens used in combination with PD-1 inhibitors were cisplatin combined with paclitaxel analogues (paclitaxel, albumin paclitaxel, paclitaxel liposomes) or combined with fluorouracil (FP), with cisplatin combined with paclitaxel analogues (TP) as the main regimen.

We first compared the PFS and OS between the two treatment groups. Additionally, we performed univariate and multivariate Cox regression analyses on different subgroups within the PC group to further explore independent risk factors that may affect patients’ PFS and OS.

## Efficacy assessment

Clinical efficacy was evaluated according to the RECIST 1.1 criteria. Clinical efficacy evaluation included complete response (CR), partial response (PR), stable disease (SD), and progressive disease (PD). The primary endpoint of the study was PFS. Secondary endpoints included objective response rate (ORR), disease control rate (DCR), OS, and safety. PFS was defined as the time from the start of treatment to disease progression or patient death from any cause. OS was defined as the time from the start of treatment to death, or the last follow-up. ORR was defined as the proportion of patients achieving CR or PR, and DCR was defined as the proportion of patients achieving CR, PR, or SD. Adverse events (AEs) were assessed according to the Common Terminology Criteria for Adverse Events (CTCAE), version 5.0.

## Statistical analysis

The chi-square test or Fisher’s exact test was used to compare the baseline characteristics between the two groups; the Kaplan-Meier curve was employed for survival analysis, with comparisons across groups using the log rank test. Univariate and multivariate Cox regression were used to analyze the predictive factors of survival; variables with a p-value less than 0.100 in univariate analysis were included in multivariate analysis. A p-value less than 0.050 was considered statistically significant. Statistical analysis was performed using IBM SPSS 26.0 and GraphPad Prism 8.0.

## Results

### Patient characteristics

A total of 278 patients receiving PD-1 inhibitors combined with chemotherapy (PC group) were included in the study. The baseline characteristics of the patients are shown in **Table 1**. The median age of the patients was 60 years (range, 41–78 years), with 91.0% being male. Patients aged 65 or older accounted for 33.5% of the total, and 92.1% had an Eastern Cooperative Oncology Group performance status (ECOG PS) score of 0-1. Patients with poorly differentiated tumors accounted for 20.5%, while those with two or more metastatic organs accounted for 51.8%. The occurrence of lymph node, lung, liver, and bone metastases was 88.8%, 23.0%, 26.3%, and 7.9%, respectively. In the treatment regimen, the PD-1 inhibitor with the highest proportion was camrelizumab (46.4%), followed by sintilimab (22.7%), pembrolizumab (15.8%), tislelizumab (11.5%), nivolumab (1.8%), and toripalimab (1.8%). The baseline characteristics, including age, sex, ECOG PS score, and number of metastatic organs, were well balanced between the C group and the PC group. However, the number of liver metastasis patients in the PC group was higher than that in the C group (*P* = 0.002) (**Table 2**).

**Table 1:** Clinical characteristics of the study population.

Characteristics	N (%)
Age (years)	
Median (range)	60 (41–78)
<65	185 (66.5)
≥65	93 (33.5)
Sex	
Male	253 (91.0)
Female	25 (9.0)
ECOG-PS	
0	13 (4.7)
1	243 (87.4)
2	22 (7.9)
Histological grade	
Well or moderately differentiated	95 (34.2)
Poorly differentiated	57 (20.5)
Indeterminate	126 (45.3)
No. of organs with metastases	
1	134 (48.2)
≥2	144 (51.8)
Lymph node Metastasis	
YES	247 (88.8)
NO	31 (11.2)
Lung Metastasis	
YES	64 (23.0)
NO	214 (77.0)
Liver Metastasis	
YES	73 (26.3)
NO	205 (73.7)
Bone Metastasis	
YES	22 (7.9)
NO	256 (92.1)
Prior radiation therapy	
YES	65 (23.4)
NO	213 (76.6)
Previous esophageal cancer surgery	
YES	91 (32.7)
NO	187 (67.3)
Palliative radiotherapy	
YES	87 (31.3)
NO	191 (68.7)
Cigarette use	
Never a smoker	124 (44.6)
Former/Current smoker	154 (55.4)
Alcohol use	
Never a drinker	119 (42.8)
Former/Current drinker	159 (57.2)
PD-L1 expression	
CPS <1	11 (4.0)
CPS ≥1	39 (14.0)
CPS <10	29 (10.4)
CPS ≥10	21 (7.6)
unknown	228 (82.0)
PD-1 blockades	
Pembrolizumab	44 (15.8)
Sintilimab	63 (22.7)
Camrelizumab	129 (46.4)
Tislelizumab	32 (11.5)
Nivolumab	5 (1.8)
Toripalimab	5 (1.8)
Chemotherapy regimen	
TP	237 (85.3)
PF	41 (14.7)
BMI	
18.5–23.9	138 (49.6)
<18.5	26 (9.4)
24.0-27.9	59 (21.2)
≥28.0	14 (5.0)
unknown	41 (14.7)
LDH	
<ULN	156 (56.1)
≥ULN	53 (19.1)
unknown	69 (24.8)
Albumin	
<35	14 (5.0)
≥35	197 (70.9)
unknown	67 (24.1)
NLR	
>3.2	112 (40.3)
≤3.2	109 (39.2)
unknown	57 (20.5)
PLR	
>183	111 (39.9)
≤183	110 (39.6)
unknown	57 (20.5)
LMR	
>2.8	108 (38.8)
≤2.8	113 (40.6)
unknown	57 (20.5)

ECOG-PS, Eastern Cooperative Oncology Group performance status; PD-L1, programmed cell death-ligand 1; CPS, combined positive score; PD-1, programmed cell death protein 1; TP, cisplatin+paclitaxel; PF, cisplatin+fluorouracil; BMI, body mass index; LDH, lactate dehydrogenase; ULN, upper limit of normal; NLR, neutrophil-to-lymphocyte ratio; PLR, platelet-to-lymphocyte ratio; LMR, lymphocyte-to-monocyte ratio.

**Table 2:** Comparison of clinical characteristics between patients treated with chemotherapy alone and PD-1 inhibitor plus chemotherapy.

Characteristics	C group n=144	PC group n=278	p value
Age			
<65	185 (66.5)	94 (65.3)	0.794
≥65	93 (33.5)	50 (34.7)	
Sex			
male	253 (91.0)	127 (88.2)	0.360
female	25 (9.0)	17 (11.8)	
ECOG-PS			
0	13 (4.7)	5 (3.5)	0.601
1	243 (87.4)	124 (86.1)	
2	22 (7.9)	15 (10.4)	
Histological grade			
Well or moderately differentiated	95 (34.2)	45 (31.3)	0.833
Poorly differentiated	57 (20.5)	31 (21.5)	
Indeterminate	126 (45.3)	68 (47.2)	
No. of organs with metastases			
1	134 (48.2)	83 (57.6)	0.066
≥2	144 (51.8)	61 (42.4)	
Lymph node Metastasis			
YES	247 (88.8)	131 (91.0)	0.499
NO	31 (11.2)	13 (9.0)	
Lung Metastasis			
YES	64 (23.0)	31 (21.5)	0.728
NO	214 (77.0)	113 (78.5)	
Liver Metastasis			
YES	73 (26.3)	19 (13.2)	0.002
NO	205 (73.7)	125 (86.8)	
Bone Metastasis			
YES	22 (7.9)	18 (12.5)	0.127
NO	256 (92.1)	126 (87.5)	
Prior radiation therapy			
YES	65 (23.4)	22 (15.3)	0.051
NO	213 (76.6)	122 (84.7)	
Previous esophageal cancer surgery			
YES	91 (32.7)	53 (36.8)	0.403
NO	187 (67.3)	91 (63.2)	
Cigarette use			
Never a smoker	124 (44.6)	68 (47.2)	0.609
Former/Current smoker	154 (55.4)	76 (52.8)	
Alcohol use			
Never a drinker	119 (42.8)	69 (47.9)	0.317
Former/Current drinker	159 (57.2)	75 (52.1)	
PD-L1 expression			
CPS <1	11 (4.0)	3 (2.1)	< 0.001
CPS ≥1	39 (14.0)	6 (4.2)	
CPS <10	29 (10.4)	4 (2.8)	
CPS ≥10	21 (7.6)	5 (3.5)	
unknown	228 (82.0)	135 (93.8)	

PD-1, programmed cell death protein 1; C group, chemotherapy alone group; PC group, PD-1 inhibitor combined chemotherapy group; ECOG-PS, Eastern Cooperative Oncology Group performance status; PD-L1, programmed cell death-ligand 1; CPS, combined positive score.

### Efficacy

**Figure 2:**
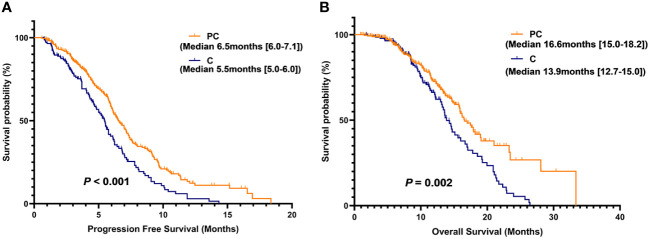
Kaplan-Meier (KM) curves for PFS **(A)** and OS **(B)** of patients in the PC group versus the C group.

**Table 3** summarizes the treatment responses of the two groups. In the C group, no patients achieved CR, 37 patients achieved PR (25.7%), 89 patients achieved SD (61.8%), 18 patients achieved PD (12.5%), ORR was 25.7%, and DCR was 87.5%. In the PC group, 3 patients achieved CR (1.1%), and 121 patients achieved PR (43.5%). The ORR and DCR were 44.6% and 91.0%, respectively. The median PFS was 5.5 months (95% CI 5.0–6.0) in the C group and 6.5 months (95% CI 6.0–7.1) in the PC group. The difference in median PFS between the two groups was statistically significant (*P <*0.001). Regarding OS, the median OS was 13.9 months (95% CI 12.7–15.0) in the C group and 16.6 months (95% CI 15.0–18.2) in the PC group. The difference in median OS between the two groups was also statistically significant (*P* = 0.002) (**Figure 2**).

**Table 3:** Subgroup analysis of overall survival by monotherapy and combination therapy.

Tumor response data	Overall population n=422	C group n=144	PC group n=278
CR	3	0	3
PR	158	37	121
SD	218	89	129
PD	43	18	25
ORR (%)	38.2%	25.7%	44.6%
DCR (%)	89.8%	87.5%	91.0%

C group, chemotherapy alone group; PC group, PD-1 inhibitor combined chemotherapy group; CR, complete response; PR, partial response; SD, stable disease; PD, progressive disease; ORR, overall response rate; DCR, disease control rate.

### Analysis of prognostic factors

We further evaluated which factors affect the prognosis of patients in the PC group through univariate and multivariate Cox model analyses. The variables with a p value <0.100 in the univariate analysis were included in the multivariate analysis. In multivariate analysis, ECOG PS score ≥1 was found to be an adverse prognostic factor for PFS (HR = 3.45, 95% CI: 1.06–11.28; *P* = 0.040). Additionally, having two or more metastatic organs (HR = 1.56, 95% CI: 1.00–2.43; *P* = 0.050) was also a negative predictor for PFS. Furthermore, the different chemotherapy regimens combined with PD-1 inhibitors were independent factors influencing patient PFS (**Table 4**). In OS multivariate analysis, bone metastasis (HR = 3.38, 95% CI: 1.42-8.02; *P* = 0.006), BMI <18.5 kg/m^2^ (HR = 2.79, 95% CI: 1.21-6.38; *P* = 0.015), LMR ≤2.8 (HR = 2.65, 95% CI: 1.13-6.21; *P* = 0.024) were adverse prognostic factors, and BMI between 24.0-27.9 kg/m^2^ (HR = 0.21, 95% CI: 0.05-0.96; *P* = 0.044) was a favorable prognostic factor (**Table 5**).

**Table 4:** Univariate and multivariate survival analyses of clinical indicators to predict risk of disease progression.

Variables	Univariate survival analyses			Multivariate survival analyses		
	HR	(95%CI)	P value	HR	(95%CI)	P value
Age (years)						
<65	1					
≥65	1.13	(0.82-1.54)	0.465			
Sex						
male	1					
female	1.61	(0.97-2.66)	0.064	1.27	(0.70-2.30)	0.438
ECOG-PS						
0	1					
≥1	1.90	(0.89-4.06)	0.096	3.45	(1.06-11.28)	0.040
Histological grade						
Well or moderately differentiated	1					
Poorly differentiated	1.10	(0.74-1.65)	0.637			
No. of organs with metastases						
1	1					
≥2	1.51	(1.12-2.03)	0.007	1.56	(1.00-2.43)	0.050
Lymph node Metastasis						
NO	1					
YES	0.82	(0.52-1.30)	0.402			
Lung Metastasis						
NO	1					
YES	1.21	(0.86-1.69)	0.272			
Liver Metastasis						
NO	1					
YES	1.47	(1.06-2.03)	0.020	1.26	(0.80-2.00)	0.320
Bone Metastasis						
NO	1					
YES	1.36	(0.84-2.22)	0.216			
Cigarette use						
Never a smoker	1					
Former/Current smoker	0.91	(0.68-1.22)	0.530			
Alcohol use						
Never a drinker	1					
Former/Current drinker	0.82	(0.61-1.11)	0.194			
PD-L1 expression						
Negative	1					
Positive	1.81	(0.61-5.35)	0.287			
PD-1 blockades						
Pembrolizumab	1					
Sintilimab	0.81	(0.50-1.32)	0.396			
Camrelizumab	0.79	(0.52-1.22)	0.286			
Tislelizumab	0.66	(0.37-1.17)	0.154			
Toripalimab	0.56	(0.19-1.68)	0.303			
Nivolumab	0.36	(0.09-1.50)	0.161			
Chemotherapy regimen						
TP	1					
PF	1.59	(1.06-2.40)	0.026	1.86	(0.15-3.01)	0.012
BMI						
18.5–23.9 kg/m^2^	1					
<18.5 kg/m^2^	1.95	(1.19-3.20)	0.008	1.47	(0.84-2.49)	0.185
24.0-27.9 kg/m^2^	0.95	(0.64-1.41)	0.802	0.98	(0.63-1.52)	0.913
≥28.0 kg/m^2^	1.36	(0.66-2.82)	0.408	1.64	(0.72-3.73)	0.240
LDH						
<ULN	1					
≥ULN	1.39	(0.95-2.04)	0.094	1.32	(0.86-2.05)	0.206
Albumin						
<35	1					
≥35	0.86	(0.38-1.95)	0.712			
NLR						
>3.2	1					
≤3.2	0.74	(0.52-1.03)	0.073	0.91	(0.60-1.38)	0.650
PLR						
>183	1					
≤183	0.89	(0.64-1.25)	0.497			
LMR						
>2.8	1					
≤2.8	1.51	(1.08-2.12)	0.016	1.20	(0.80-1.81)	0.387

HR, hazard ratio; CI, confidence interval; ECOG-PS, Eastern Cooperative Oncology Group performance status; PD-L1, programmed cell death-ligand 1; PD-1, programmed cell death protein 1; TP, cisplatin+paclitaxel; PF, cisplatin+fluorouracil; BMI, body mass index; LDH, lactate dehydrogenase; ULN, upper limit of normal; NLR, neutrophil-to-lymphocyte ratio; PLR, platelet-to-lymphocyte ratio; LMR, lymphocyte-to-monocyte ratio.

**Table 5:** Univariate and multivariate survival analyses of clinical indicators to predict risk of death.

Variables	Univariate survival analyses			Multivariate survival analyses		
	HR	(95%CI)	P value	HR	(95%CI)	P value
Age (years)						
<65	1					
≥65	1.09	(0.70-1.70)	0.702			
Sex						
male	1					
female	1.11	(0.54-2.32)	0.772			
ECOG-PS						
0	1					
≥1	1.15	(0.42-3.13)	0.790			
Histological grade						
Well or moderately differentiated	1					
Poorly differentiated	0.60	(0.33-1.10)	0.098	0.85	(0.35-2.06)	0.722
No. of organs with metastases						
1	1					
≥2	1.01	(0.67-1.52)	0.950			
Lymph node Metastasis						
NO	1					
YES	1.09	(0.55-2.181)	0.800			
Lung Metastasis						
NO	1					
YES	0.77	(0.46-1.31)	0.335			
Liver Metastasis						
NO	1					
YES	1.24	(0.79-1.95)	0.359			
Bone Metastasis						
NO	1					
YES	1.89	(0.97-3.65)	0.060	3.38	(1.42-8.02)	0.006
Cigarette use						
Never a smoker	1					
Former/Current smoker	0.92	(0.61-1.40)	0.699			
Alcohol use						
Never a drinker	1					
Former/Current drinker	0.86	(0.57-1.30)	0.477			
Prior radiation therapy						
YES	1					
NO	0.65	(0.43-1.00)	0.048	0.75	(0.33-1.70)	0.489
Palliative radiotherapy						
YES	1					
NO	2.03	(1.27-3.23)	0.003	1.47	(0.65-3.33)	0.357
PD-L1 expression						
Negative	1					
Positive	2.23	(0.28-17.63)	0.448			
PD-1 blockades						
Pembrolizumab	1					
Sintilimab	0.82	(0.42-1.57)	0.524			
Camrelizumab	0.93	(0.53-1.62)	0.787			
Tislelizumab	0.99	(0.50-2.00)	0.991			
Toripalimab	0.49	(0.11-2.14)	0.342			
Nivolumab	0.83	(0.20-3.59)	0.800			
Chemotherapy regimen						
TP	1					
PF	1.26	(0.72-2.18)	0.422			
BMI						
18.5–23.9 kg/m^2^	1					
<18.5 kg/m^2^	1.74	(0.92-3.30)	0.090	2.79	(1.22-6.38)	0.015
24.0-27.9 kg/m^2^	0.51	(0.43-2.46)	0.045	0.21	(0.05-0.96)	0.044
≥28.0 kg/m^2^	1.03	(0.26-0.100)	0.944	0.50	(0.06-4.12)	0.519
LDH						
<ULN	1					
≥ULN	0.78	(0.42-1.43)	0.418			
Albumin						
<35	1					
≥35	0.37	(0.13-1.03)	0.056	0.56	(0.16-1.99)	0.368
NLR						
>3.2	1					
≤3.2	0.68	(0.42-1.10)	0.117			
PLR						
>182.4	1					
≤182.4	0.82	(0.50-1.33)	0.422			
LMR						
>2.8	1					
≤2.8	1.79	(1.09-2.94)	0.022	2.65	(1.13-6.21)	0.024

HR, hazard ratio; CI, confidence interval; ECOG-PS, Eastern Cooperative Oncology Group performance status; PD-L1, programmed cell death-ligand 1; PD-1, programmed cell death protein 1; TP, cisplatin+paclitaxel; PF, cisplatin+fluorouracil; BMI, body mass index; LDH, lactate dehydrogenase; ULN, upper limit of normal; NLR, neutrophil-to-lymphocyte ratio; PLR, platelet-to-lymphocyte ratio; LMR, lymphocyte-to-monocyte ratio.

**Figure 3:**
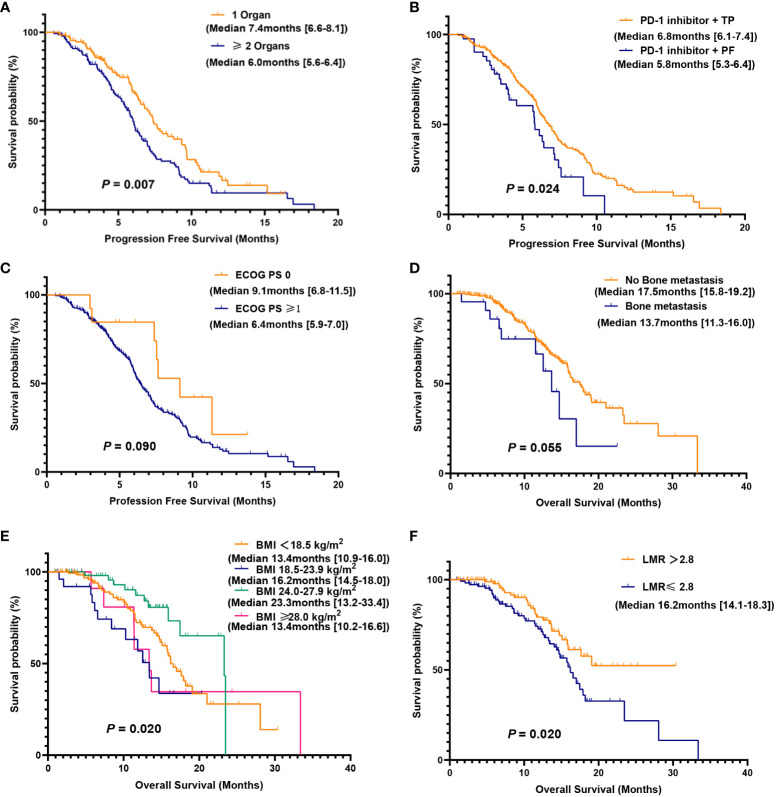
Kaplan-Meier (KM) curves for PFS and OS were analyzed for subgroups of the PC group. PFS **(A)** in patients with 1 or ≥2 metastatic organs; PFS **(B)** of PD-1 inhibitor combined with TP or PF; PFS **(C)** of different ECOG PS score; OS **(D)** in patients with or without bone metastasis; OS **(E)** for patients with different BMI; OS **(F)** for patients with low and high LMR.

Based on the results of the multivariate Cox model analysis, further survival analysis was conducted. The median PFS was significantly different in the subgroup of patients with 1 or ≥2 metastatic organs (median PFS: 7.4 vs. 6.0 months, *P* = 0.007). The difference in median PFS between the PD-1 inhibitor plus TP regimen and the PD-1 inhibitor plus PF regimen was also statistically significant (median PFS: 6.8 vs. 5.8 months, *P* = 0.024), while there was no significant difference in ECOG PS score of 0-1 (median PFS: 9.1 vs. 6.4 months, *P* = 0.090). There was no significant difference in median OS between patients without and with bone metastases (17.5 vs. 13.7 months, *P* = 0.055), but there was a trend toward a significant difference. Compared with patients with BMI <18.5 kg/m^2^, BMI 18.5-23.9 kg/m^2^, and BMI ≥28.0 kg/m^2^, patients with BMI 24.0-27.9 kg/m^2^ had the longest median OS (median OS: 13.4 vs. 16.2 vs. 13.4 vs. 23.3 months, *P* = 0.020). There was a significant difference in median OS between patients with LMR ≤2.8 and LMR >2.8 (*P* = 0.020), while patients with LMR >2.8 did not reach the median OS (**Figures 3A–F**).

### Safety

**Table 6** shows some adverse reactions that occurred during the treatment process in the PC group. The most common adverse reactions were hematological toxicity, such as anemia (77.3%), white blood cell count reduction (53.2%), and platelet count reduction (39.9%), followed by gastrointestinal adverse reactions. There are also a few immune-related adverse reactions, such as hypothyroidism, immune-mediated lung disease, and reactive capillary hyperplasia caused by camrelizumab. Although some patients experienced grade 3 or higher adverse reactions, the overall safety was manageable, and no treatment-related deaths occurred.

**Table 6:** Adverse events in the PC group.

Treatment-related adverse events	All grades n (%)	grade ≥3 n (%)
Anemia	215 (77.3)	40 (14.4)
Decrease in white blood cell count	148 (53.2)	42 (15.1)
Decrease in neutrophil count	111 (39.9)	37 (13.3)
Decrease in platelet count	92 (33.1)	8 (2.9)
Nausea	125 (45.0)	4 (1.4)
Vomiting	82 (29.5)	5 (1.8)
Reactive capillary endothelial proliferation	81(29.1)	0 (0.0)
Hypoalbuminemia	70 (25.2)	0 (0.0)
Abnormal hepatic function	8 (2.9)	1 (0.4)
Hypothyroidism	24 (8.6)	0 (0.0)
Pruritus	15 (5.4)	1 (0.4)
Rash	9 (3.2)	0 (0.0)
Immune-mediated lung disease	3 (1.1)	1 (0.4)

## Discussion

Patients with early esophageal cancer do not have obvious symptoms, but dysphagia occurs when the esophageal mucosa has lesions and the lesions invade 2/3 of the esophagus, so about 90% of patients are diagnosed at an advanced stage, and about 50% of patients with esophageal cancer have metastatic disease at diagnosis (13). In this study, some patients did not undergo regular evaluation of their condition after early treatment. When discomfort symptoms reappeared and they were admitted for treatment, distant metastasis had already occurred. Although in clinical trials of first-line treatment for advanced ESCC, patients with distant metastasis are included in the inclusion criteria, there is a lack of independent real-world studies of patients with distant metastasis. This article further analyzes this group of patients.

In this study, PD-1 inhibitors combined with chemotherapy were compared with chemotherapy alone and patients’ median PFS and median OS (median PFS 6.5 vs. 5.5 months, *P* = < 0.001) and (median OS 16.6 vs. 13.9 months, *P* = 0.002). The PD-1 inhibitors used by the patients in this study were mainly camrelizumab and sintilimab, accounting for 69.1% of the total patients in the PC group. In the ESCORT-1st randomized controlled trial, regardless of PD-L1 expression, the combination of camrelizumab and chemotherapy compared to chemotherapy alone resulted in a median OS of 15.3 vs. 12.0 months (HR = 0.70; 95% CI: 0.56-0.88, *P* = 0.001) and a median PFS of 6.9 vs. 5.6 months (HR = 0.56; 95% CI: 0.46-0.68, *P* < 0.001) (9). In the phase III clinical trial ORIENT-15, for the overall population, the combination of sintilimab and chemotherapy significantly extended the median OS by 4.6 months (median OS 17.4 months vs. 12.8 months) and reduced the risk of death by 33.9% (HR = 0.661, *P* < 0.001). The median PFS was 7.2 vs. 5.7 months (HR = 0.56, 95% CI: 0.46 to 0.68, *P* < 0.001) (8). Although all the included patients developed distant metastatic disease, some patients only developed oligometastases. The patients’ basic physical condition and treatment tolerance were relatively good, and some patients took palliative radiotherapy for bone and brain metastases. Some patients’ esophageal obstruction aggravated in the later stage of the disease using stent placement or palliative radiotherapy to relieve their symptoms. These may be the reasons for the further improvement in the survival prognosis of the patients in this study. In a study, further analysis was conducted on the use of stent placement or palliative radiation therapy for the treatment of swallowing difficulties and pain in patients with metastatic esophageal cancer. Compared to esophageal stent placement, palliative radiation therapy provided rapid and sustained relief of pain (*P* < 0.001) and had lower occurrence rates of esophageal fistula and bleeding. Although stent placement showed greater and faster improvement in swallowing difficulties compared to palliative radiation therapy, there was no significant difference in the improvement of swallowing difficulties over time between the two treatment groups for patients with a pre-intervention swallowing difficulty score of ≥2 (14).

There is no consensus on the optimal first-line chemotherapy regimen for patients with advanced or metastatic ESCC worldwide (15). The chemotherapy regimens used in clinical practice in different regions are also not unified. In China, the TP regimen is mainly used, while in other countries, the PF regimen is more popular (16). The results of this study showed that the median PFS of patients with PD-1 inhibitors combined with the TP regimen was more than one month longer than that of patients with the combined PF regimen, and there was statistical significance. In a retrospective study on the first-line treatment of locally advanced or advanced ESCC with or without TP and PF combined with immune checkpoint inhibitors (ICIs), it was found that the ORR and DCR in the TP+ICIs group were 42.1% (50/119) and 97.5% (116/119), respectively, which were 6.6% and 7.2% higher than those in the PF+ICIs group. Regardless of whether the TP regimen is combined with ICI or not, the PFS and OS benefits of the TP regimen are significantly better than those of the PF regimen (16). Related studies have shown that in clinical trials, the ORR of patients receiving PD-1 inhibitors combined with TP or FP seems to vary, possibly due to different chemotherapy regimens that may cause different changes in the tumor microenvironment. For example, paclitaxel can induce the immunogenic cell death of cancer cells and cause various immunogenic effects (17). Preclinical studies have also shown that fluorouracil drugs and paclitaxel drugs have different effects on dendritic cell maturation and the elimination of myeloid inhibitory cells in the tumor microenvironment, which may affect T cell-dependent anti-tumor responses (18, 19). For esophageal cancer, most of them affect the diet of patients, leading to reduced food intake. At the same time, the tumor also increases energy consumption. The final results in this study indicated that the survival prognosis of overweight patients with a BMI between 24.0 and 27.9 kg/m^2^ was better than that of other groups, while the median OS of patients with a BMI below 18.5 kg/m^2^ was significantly lower than that of other groups. Multivariate Cox analysis also demonstrated that BMI could be used as a predictor of survival for ESCC with distant metastasis. In a retrospective study of 615 consecutive Chinese esophageal cancer patients who underwent tube resection and/or chemotherapy/radiotherapy, it was found that the 10-year OS of patients with high BMI was significantly longer than that of patients with low BMI. Pretreatment patients with a high BMI and no decrease in BMI have increased overall survival (20). The same results were obtained in this study, but we further refined the patients with high BMI into overweight and obese patients. In the multivariate Cox analysis of PFS in this study, a higher ECOG PS score and more metastatic organs were negative predictors for PFS, but BMI was not statistically significant, which may indicate that the patient’s physical and functional conditions before treatment determine the tolerance and response to a PD-1 inhibitor combined with chemotherapy. BMI has better predictive power for the long-term survival of patients.

Research has shown that inflammation is closely related to the occurrence, progression, and metastasis of tumors, and systemic inflammatory factors are independent risk factors for the formation and development of various solid tumors (21–23). Neutrophils can promote tumor progression by inhibiting adaptive immune responses in the tumor microenvironment, and an increase in neutrophil count may have adverse effects on tumor patients (24). Lymphocytes play an important role in tumor-related immunity and can inhibit the progression of various tumors. A decrease in lymphocyte count indicates that the body is in an immunosuppressive state (25). Some current studies have shown that low LMR is associated with low survival in a variety of cancers, such as breast, ovarian, and hepatocellular carcinoma (26–28). The results of this study also confirmed that the median OS of the high LMR group was significantly higher than that of the low LMR group, and the high LMR group can serve as a positive predictive factor for patient OS. Liu et al. used LMR to predict tumor response and survival in patients with locally advanced esophageal cancer who received definitive radiotherapy and found that there were more patients with CR in the high LMR group (36/48, *P* < 0.001) and that the CR group had a higher LMR value than the non-CR group (4.89 ± 1.17 vs. 3.87 ± 1.29, *P* < 0.001) (29). In a meta-analysis, 1701 patients with esophageal squamous carcinoma from seven studies were included to assess the prognostic value of preoperative LMR in ESCC, and the analysis concluded that low LMR was associated with advanced clinicopathological features and poor prognosis in patients with ESCC and could be used as a predictive biomarker for patients with ESCC (30). However, the relationship between NLR, PLR, and survival prognosis was not confirmed in the results of the present study, which may be related to the selection of cut-off values and the need to further expand the sample size for analysis.

Compared with early cancer patients, patients with distant metastasis have a poorer survival rate and are the main cause of cancer-related deaths. Esophageal cancer is most commonly spread to organs such as the lymph nodes, lungs, liver, and bones. In this study, bone metastasis was found to be a negative predictor of patient survival and prognosis. Shi et al. also found that bone metastasis (OR, 2.60; 95%CI, 1.65–4.11; *P* < 0.001) is an independent risk factor for early death in untreated patients with stage IV esophageal cancer (31). Qiu et al. included 855 elderly patients with stage IVB esophageal adenocarcinoma who developed distant metastasis and found that patients with single-organ bone metastasis had the worst OS and cancer-specific survival among patients with single-organ metastasis (32).

The adverse reactions that occur during the treatment process mainly include hematological toxicity and gastrointestinal reactions. The use of PD-1 monoclonal antibodies will also cause immune-related adverse reactions. The overall adverse reactions were controllable. The occurrence and development of adverse reactions can be well controlled through temporary cessation of medication, reduction of dosage, and timely symptomatic treatment.

Our research has some limitations. Firstly, this is a retrospective study with potential information bias. For example, due to the fact that some PD-1 inhibitors have just been approved for use in China, the number of patients included in each PD-1 inhibitor was uneven, and the sample size was relatively small, which may affect the generalizability of the results. Furthermore, due to economic reasons, many patients in this study did not undergo PD-L1 immunohistochemical testing, making it impossible to compare subgroups based on different PD-L1 immunohistochemical results. We chose the median values of NLR, PLR, and LMR as the cutoff values. Although this method can minimize false-positive results, it is currently unclear whether different critical values or thresholds will affect the prediction of tumor recurrence and survival in patients. In addition, some medical records were incomplete, and a lack of data leads to incomplete data collection. Based on these limitations of this study, further validation can be achieved by conducting large-scale prospective studies to obtain more accurate research results.

## Conclusion

For ESCC patients with distant metastasis, the combination of PD-1 inhibitors and chemotherapy in the real world has good clinical effects, survival prognosis, and safety compared to clinical trial studies. Further large-scale research is needed to verify the impact of different factors on patient survival.

## Data availability statement

The raw data supporting the conclusions of this article will be made available by the authors, without undue reservation.

## Ethics statement

The studies involving humans were approved by Ethics Committee of Shandong Cancer Hospital and Institute. The studies were conducted in accordance with the local legislation and institutional requirements. Written informed consent for participation was not required from the participants or the participants’ legal guardians/next of kin in accordance with the national legislation and institutional requirements.

## Author contributions

LG: Data curation, Formal analysis, Writing – original draft, Methodology, Software, Writing – review & editing. LT: Data curation, Formal analysis, Writing – review & editing. JP: Data curation, Formal analysis, Writing – review & editing. ZH: Data curation, Software, Writing – review & editing. JY: Project administration, Writing – review & editing. BL: Methodology, Supervision, Writing – review & editing.
